# Chronic meningococcemia in a 16-year-old boy: a case report

**DOI:** 10.4076/1757-1626-2-7103

**Published:** 2009-07-31

**Authors:** Solen Kernéis, Emmanuel Mahé, Beate Heym, Valérie Sivadon-Tardy, Françoise Bourgeois, Thomas Hanslik

**Affiliations:** 1 AP-HP, Service de Médecine Interne, Hôpital Ambroise ParéF-92100, Boulogne BillancourtFrance; 2 AP-HP, Service de Dermatologie, Hôpital Ambroise ParéF-92100, Boulogne BillancourtFrance; 3 Université Versailles Saint-Quentin-en-YvelinesF-78000, VersaillesFrance; 4 AP-HP, Service de Microbiologie, Hôpital Ambroise ParéF-92100, Boulogne BillancourtFrance

## Abstract

Herein, we present a case of meningococcal disease in a patient presenting with of a three-week history of fever, cutaneous vasculitis and joint pain, in whom chronic meningococcemia was retained as presumptive diagnosis, after the disease evolved towards meningitis. This unusual case illustrates the great heterogeneity in possible clinical presentations of *Neisseria meningitidis* infections and underlines that diagnosis should always be evocated when facing the triad of fever, vasculitic skin eruption and big joints arthralgia, in a person in otherwise good general condition.

## Case presentation

A 16 year-old French Caucasian boy was referred to our hospital for investigation of a three-week history of fever associated with cutaneous lesions.

He had no remarkable personal or familial past medical history, and was not taking any regular medication. The illness had begun with chills, fever up to 40.5°C and night sweats. Approximately seven days after the onset of the disease, he reported a skin eruption. The patient did not report any headaches, cough, rhinorrhea, signs of urinary tract infection, testis pain, nor ophtalmologic disorders. There was no evidence for arthropod bite, prior sexual contact, or contact with ill persons.

**Figure 1.**
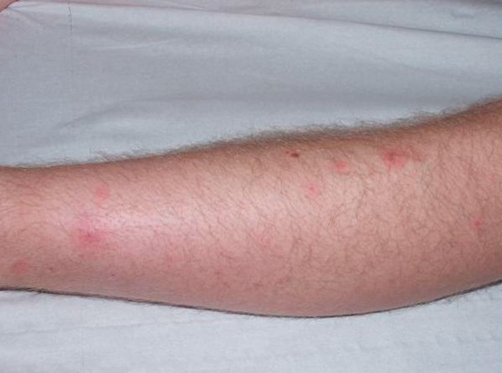
Inflammatory papules of the leg.

**Figure 2.**
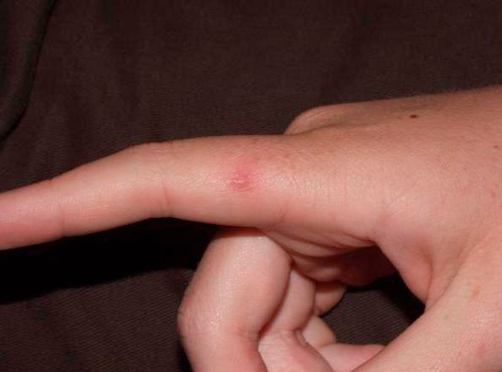
Pustules of the index finger.

**Figure 3.**
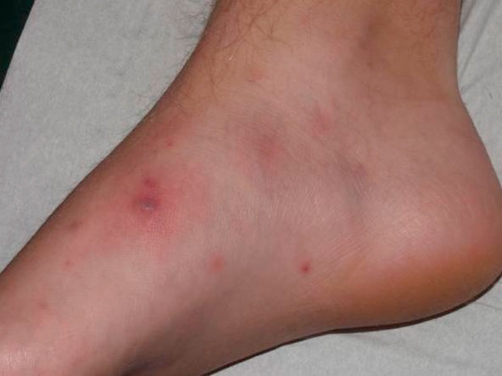
Pustules of the foot.

At admission, the patient was in good physical condition, and was perfectly alert and orientated. His temperature was 37°C, the blood pressure was at 110/70 mmHg and pulse rate was at 88 per minute. Cutaneous lesions on the forearms and the legs consisted in pustules and inflammatory papules (Figure 1, Figure 2 and Figure 3). The nasal and pharyngeal mucosa was normal. Cardiac, chest, upper respiratory tract, abdominal and neurological examinations were unremarkable.

**Figure 4.**
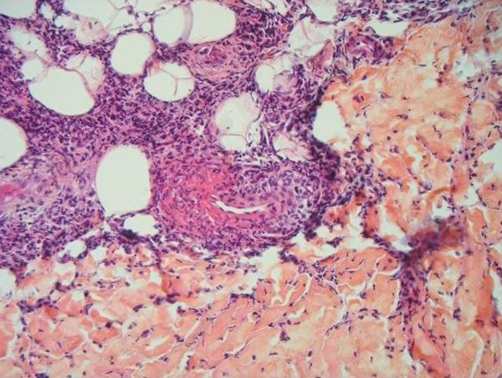
Histology of the skin biopsy showing unspecific leukocytoclastic vasculitis.

The blood cell counts were normal. The urine and blood cultures were negative. The C-reactive protein was elevated at 70 mg/L (normal <5 mg/L). The chest X-ray, the electrocardiogram and the abdominal ultrasonography were normal. A skin biopsy showed unspecific leukocytoclastic vasculitis with small lymphocytes and neutrophils (Figure 4). Microscopy after Gram staining was negative.

No etiology could be evoked for this febrile rash. Since the patient was in good condition, he was discharged with a symptomatic medication against pain and fever (Paracetamol). He was advised to check carefully his temperature and to come back in case of unfavourable evolution. Within 48 h, the patient was readmitted at the hospital because of violent frontal headaches and vomiting. On examination he appeared acutely ill and lethargic with fever up to 39°C and marked neck stiffness. He was immediately treated with intravenous ceftriaxone and referred to the intensive care unit where a lumbar puncture was performed. The cerebrospinal fluid (CSF) examination disclosed 1,124 white blood cells/mL (99% polymorphonuclear neutrophils), proteins were at 2.14 g/L and glucose 1.6 mmol/L. Gram staining revealed Gram-negative diplococci, but culture remained negative, most probably due to the antibiotic treatment prior to the lumbar puncture. The patient fully recovered within two days and ceftriaxone therapy was followed with a 5-day course of intravenous amoxicillin. He was discharged from the hospital seven days after admission.

## Discussion

In the present case report, the three-week history of febrile illness was ascribed to chronic meningococcemia after the disease evolved towards meningococcal meningitis. The diagnosis of meningococcal meningitis was assessed on the basis of the neurological febrile syndrome with the intense pleiocytosis of the CSF and the presence of Gram-negative diplococci at direct microscopic examination, despite the lack of positive culture.

Here, differential diagnosis consisted primarily in disseminated gonococcal infection. It is responsible of skin lesions of varying size and stages of development, often starting as macules and progressing into papules, vesicopustules, and bullae, sometimes necrotic [1], similar to the lesions of the case reported here. In rare occasions, it may involve the meninges. However, this patient had no frank arthralgia nor tenosynovitis which are frequent findings in disseminated gonococcal infection, and above all he negated any prior sexual contact.

Chronic (or benign) meningococcemia is a very rare form of meningococcal infection. When searching the Medline database, only seldom cases have been reported in the last 40 years. It is defined as a meningococcal sepsis of at least one week's duration without meningeal symptoms and is characterized by a prolonged clinical course with intermittent fever, rash and migratory arthralgia [2], usually occurring in healthy children or young adults. Fever can be spiking, persisting or relapsing. Skin eruption is seen in over 90% of cases and consists in erythematous macules evolving into tender nodules which become frankly purpuric, sometimes necrotic. Arthralgia are reported in 70% of cases and involve mostly the big joints [2,3,4]. The diagnostic is challenging as bacterial cultures are frequently negative in the initial stages of the illness [2,3]. Chronic meningococcemia may be self-limiting, but meningitis and death can occur as a late complication [2,3]. The pathophysiology of chronic meningococcemia remains unclear. *N. meningitidis* of serogroup B seems to be more often involved than other serogroups [5,6]. Some authors state that the relatively reduced virulence of serogroup B may partly explain the chronicity of clinical signs and the reduced inflammatory reponse [6]. Host factors are also important. Indeed, even in the absence of mass-vaccination, a majority of adults after 25 years of age are fully immunised against most serotypes of *N. meningitidis*, leading to very few acute meningococcemia diseases in immunocompetent adults. Thus, the relatively moderate clinical signs of chronic meningococcemia may be explained by a pre-existent immunity against *N. meningitidis*.

This case report highlights the heterogeneity in possible clinical presentations of meningoccal disease. The diagnosis of chronic meningococcemia should always be evocated when facing the triad of spiking fever, vasculitic skin eruption and big joints arthralgia, in a person in otherwise good general condition. It should prompt to consider appropriate antibiotic treatment so as to avoid possible severe complications.

## Abbreviation

CSF: Cerebrospinal Fluid
